# Delay in treatment initiation and its association with clinical severity and infectiousness among new adult pulmonary tuberculosis patients in Tigray, northern Ethiopia

**DOI:** 10.1186/s12879-020-05191-4

**Published:** 2020-06-29

**Authors:** Kiros Tedla, Girmay Medhin, Gebretsadik Berhe, Afework Mulugeta, Nega Berhe

**Affiliations:** 1 Institute of Biomedical Science, College of Health Science, Mekelle University, Mekelle, Ethiopia; 2 Aklillu Lema Institute of Pathobiology, Addis Ababa University, Addis Ababa, Ethiopia; 3 School of Public Health, College of Health Science, Mekelle University, Mekelle, Ethiopia

**Keywords:** Tuberculosis, Clinical severity, Risk level of infectiousness, Cross-sectional, Tigray, Ethiopia

## Abstract

**Background:**

Delayed treatment initiation of tuberculosis (TB) increases disease progression and development of complications which may lead to a higher level of infectiousness, clinical severity and increased mortality. But published evidences that investigated the effect of delayed initiation of treatment on clinical severity and level of infectiousness of pulmonary tuberculosis patients is scarce in Tigray, Northern Ethiopia.

**Objective:**

To investigate the association of delayed treatment initiation of new adult Pulmonary Tuberculosis patients with clinical severity and level of infectiousness.

**Methods:**

In this cross-sectional study design, a total of 875 newly diagnosed adult pulmonary tuberculosis patients were recruited from 21 health facilities from October 2018 to October 2019. Health facilities and study participants were selected by a simple random sampling method. Data were collected using questionnaires through face-to-face interviews of patients within the first 2 weeks of treatment initiation. Clinical severity was assessed by Bandim tuberculosis score and level of infectiousness was assessed by smear positivity or lung cavitations. Data were analyzed using SPSS version 21 software program. Logistic regression analysis was used to ascertain the association of delay with clinical severity and level of infectiousness. *P*-BMC Public Health of less than 0.05 was reported as being statistically significant.

**Results:**

Those who had initiated treatment without delay and those who have initiated treatment after a medium delay of 31 to 60 days were significantly associated with decreased clinical score compared to those who initiated treatment after a delay of more than two months. Compared with patients who have initiated treatment within one month, the level of infectiousness was greater for delay of 30–60 days and above 60 days. Patients having more than 3 family members have higher level of infectiousness as compared to those who have a maximum of 3 family members. Whereas, patients having at least two rooms and being HIV negative had lower levels of infectiousness compared to their counter patients.

**Conclusion:**

Narrowing the gap between their initial occurrence of TB symptoms and treatment initiation is the way forward to improve clinical courses of TB patients and to reduce the level of infectiousness of TB to other people from these patients.

## Introduction

Tuberculosis (TB), caused by *Mycobacterium tuberculosis*, is one of the leading causes of morbidity and mortality worldwide [1, 2]. Despite the implementation of control mechanisms, TB continues to be one of the top ten causes of deaths worldwide with 4000 deaths every day and nearly 30,000 people fall ill with this disease [2].

To successfully control TB, early detection, and treatment initiation is mandatory [3]. Delay in TB treatment initiation can be due to patient delay (late presentation with symptoms), health provider delay (failure of the health system to diagnosis tuberculosis) or total treatment delay (delay due to late presentation and late diagnosis of tuberculosis) [4]. In a systematic review of delays worldwide the reported median ranges, total delay 25–185 days, patient delay 4.9–162 days, and health system delay 2–87 days for both low and high-income countries. Both patient delay and health system delay in low-income countries were 31.7 days and 28.5 days, respectively [5]. In African countries, the time from showing of signs and symptoms up to treatment initiation ranges from 40 days in Uganda [6] up to 104 days in Ghana [7].

Delayed treatment initiation of tuberculosis (TB) has the potential to increase the risk of severe disease and mortality because of disease progression and the development of complications [8]. This rapid disease progression also increases disease transmission within the community and creates a favorable condition for the TB epidemic [9, 10]. Few studies have explored the effects of treatment initiation delay of tuberculosis (TB) patients on the risk of infection among their close contacts, clinical severity, and treatment outcome [11, 12]. Studies had also reported the relationship between duration of total delay of smear-positive TB patients and TB infection with a turning point at 30 days after which the risk of TB infection and clinical severity increased significantly [4, 8, 13]. Most transmissions occur between the appearance of cough and initiation of treatment as patients become more contagious with prolonged because of the highest bacillary numbers on sputum smears [14]. There are also contradicting results showing that delay was not related to increased transmission [15, 16].

Ethiopia is among the 30 TB high burden countries worldwide [2], where TB is a major public health problem. In Ethiopia the median TB patient’s treatment initiation varies from settings to settings including 36 days in Desie [17], 28 days in Arsi [18], 90 days in Tigray [19], 97 days in East Wollega [20] and 80 days in Afar region [13].. The median health system and patient delays were 22 and, 30 days in the Amhara region [21, 22], 22 and 25 days in the southern region [23], 9, and 30 days in the Tigray region [19, 24]. However, there is limited evidence on the association of treatment initiation delay with clinical severity and level of infectiousness in Ethiopia and elsewhere. Hence, the current study aimed to investigate the association of TB delay in treatment initiation with the clinical severity and level of infectiousness. This would be important for setting interventions in the clinical setup for better management of TB patients and to successfully control TB.

## Methods

### Study area

The study was conducted in two zones (Mekelle and Eastern zones) of the Tigray Region, Northern Ethiopia. Mekelle is the Regional capital which is located 778 km from Addis-Ababa. The region has seven administrative zones: comprising a total of 53 Weredas (districts) and 673 Tibias (sub-districts). The population is above 5.3 million which is about 6% of the total population of Ethiopia. There are 712 health posts, 214 health centers and 38 hospitals (1 specialized hospital, 15 general hospitals, 22 primary hospitals) in the region. There are also well-organized community-based structures that support the health system at the grass-root level (health extension workers and women development army). The private health sector also plays a significant role in relieving the burden of the patient load from the public facilities with strong policy support [25, 26]. Currently, almost all health facilities are providing directly observed short-course tuberculosis treatment by trained health workers (TB focal persons). The program was introduced in all hospitals, health centers and in most of the health posts for the past years [25].

### Study design and period

A cross-sectional survey was conducted to investigate the association of treatment initiation delay of Pulmonary Tuberculosis (PTB) patients on clinical severity and level of infectiousness in health facilities of two zones of Tigray Region, Northern Ethiopia from October 2018 to October 2019.

### Source or target population

All new pulmonary tuberculosis patients (PTB) diagnosed and treatment initiated in all health facilities of Tigray region.

### Study population

All new pulmonary tuberculosis (PTB) cases diagnosed and treatment initiated in the health facilities of the two zones of Tigray region.

### Inclusion criteria

bacteriologically positive or x-ray positive PTB patients whose age is 18 years and above, with no previous history of TB, non-MDR-TB cases and who are diagnosed and initiated treatment within the study settings were included in the study.

### Sample size

In calculating the sample size that enable us to estimate the effect of delay on clinical severity and level of infectiousness, we considered 95% confidence interval, 80% power, an equal number of exposed and non-exposed (the delay was the exposer). The calculated sample size for the objective was 850 study participants.

### Recruitment of study participants

Out of the seven zones of the region, two zones (i.e. Eastern zone and Mekelle zone) were selected because of their proximity to the research Institute where the TB culture is performed, and logistical reasons that emanates from the need to have frequent visit to the study sites. Furthermore, the only functional specialized hospital is located in Mekelle. Within the selected zones, there were 26 health centers and 6 hospitals that reported average monthly cases of 3 and above in the years 2016/2017 and 2017/18 [25]. Hence, of the 26 health centers and 6 hospitals, 16 were selected by a simple random sampling method using the lottery method and five hospitals were included in the study excluding one hospital as it is a specialized hospital for ophthalmological service only. The calculated sample size was proportionally allocated to each health facility based on the average number of TB cases reported by the facility in the prior 2 years. Study participants within each health facility were selected using a simple random sampling technique (lottery method) by using TB register book as a sampling frame until the allocated sample size was attained.

### Data collection

The data collection tool was drafted from previously tested instruments and reviewed literature [21, 27, 28] and then modified to fit the context of the research interest. Study participants were identified during their treatment start and invited for consultation and enrolment. The samples for laboratory investigations were collected before treatment initiation. Data were collected on socio-demographic and lifestyle factors, clinical and diagnosis related factors, outcome measurement using Bandim TBscore, and measurement of the level of infectiousness as described below.

#### Socio-demographic and lifestyle data

Background data collected from study participants include gender, age, residence, occupation, marital status, education, family size, income as indebt (poor), income = expense, and saving; smoking history as smoker and non-smoker; a person was considered as a smoker if he was currently smoking or had history of smoking and alcohol intake in the last three/6 months period (Yes, No); yes were recorded if the person takes any form of alcohol (traditional or modern alcohol).

#### Clinical and diagnostics data

Clinical examination and interviews of patients were carried out at recruitment or inclusion. During this time Symptoms (respiratory symptoms, constitutional symptoms, or both), smear status (as positive and negative), pulmonary cavity by chest x-ray (yes/no), and patient delay duration by (< 30 days, 31–60 days, and > 60 days) were collected. Respiratory symptoms included dyspnea, hemoptysis, cough, or expectoration while constitutional symptoms included malaise, loss of appetite, weight loss, fatigue, fever, chills, chest pain or night sweats.

Smear microscopy of all the study participants was done at three GeneXpert sites of the study area. Three slides per individual were prepared and examined by two laboratory technologists independently. X-ray of the study participants was done in five of the study hospitals that have an X-ray machine. These results were examined by medical doctors (a medical doctor) specialized in radiology.

#### Bandim TBscore

Clinical severity was assessed by the Bandim TBscore [12]. The Bandim TBscore is a newly developed tool to assess the clinical status of patients with TB. Its value is expressed as a numeric index based on cough, hemoptysis, dyspnoea, chest pain, night sweating, anemic conjunctivae, tachycardia, positive lung auscultation, increased temperature, body mass index (BMI) and middle-upper arm circumference (MUAC). A Bandim TBscores ≥8 correlates with mortality and lower TBscore correlates with favorable outcomes, with cure and treatment success [29, 30]. In these studies and other settings [12] severe TB was defined as TBscore ≥8, which has a strong predictive capacity for mortality.

#### Scoring and severity classes

The score was based on 11 clinical variables (i.e. cough, hemoptysis, dyspnoea, chest pain and night sweating, anemic conjunctivae, tachycardia, positive lung auscultation, increased temperature, body mass index (BMI < 16 and BMI < 18) and middle-upper arm circumference (MUAC< 200 mm and MUAC< 220 mm). Each of the 11 variables is scored 0/1 point; the total score was generated resulting in the maximum score of 13 points. For all variables examined and used in the score, missing values were coded as zero. The patients were classified into two severity classes as TBscore < 8 as having less severe and TBscore having > 8 as having a higher severity class.

### Measurement of the level of infectiousness

In this study sputum smear, positivity and presence of pulmonary cavity were used as measures for the level of infectiousness of TB patients which were used as proxy measures of risk of MTB transmission [28, 31, 32]. Patients were considered as highly infectious if they were in one of the following categories: 1) being smear-positive with lung cavitations 2) Smear-positive but had no lung cavitations 3) Smear-negative but had lung cavitations. Whereas, TB patients who were smear-negative and with no lung cavitations were considered as having less level of infectiousness.

### Data quality and management

The structured questionnaire was pretested before the actual data collection. The adapted questionnaire was reviewed by experts in Tigray regional health bureau and other clinical researchers experienced in this field in order to clarify confusing items, and to comment on the apparent validity of each item. The comments from the expertise were included and the resulting questionnaire was administered to data collectors to further check items that might not be easy to understand in 50 TB patients as a pilot study. Finally the questionnaire was modified according to the results of the pilot study and the comments from the experts.

The actual data collection was done using the final version of the questionnaire by 21 nurses having a BSc degree of which three were senior supervisors. Close supervision was done together with the PI of the project. These data collectors were trained on the objectives of the study, the importance of the findings and on how to collect the data from study participants for 5 days. The training was performed through demonstration and role-playing. The questionnaire was converted into local language Tigrigna where the majority of the people speak. The entire interview was done using the local language. Finally, the interviewer checks for completeness and the data was entered into SPSS software.

During sample collection for laboratory investigation, patients were advised to submit thick sputum rather than saliva and the sputum sample was collected as per standards [27]. Before smearing, specimens were assessed macroscopically for quality and recorded as purulent with whitish color or bloody. The study numbers or codes were covered with wrap around stickers before microscopy. As part of internal quality control (IQC), all the positive slides and randomly selected 50% of negative slides were re-examined by a different laboratory technologist.

### Data analysis

Data was entered and analyzed using “SPSS version 21” [33]. The data was summarized and presented using frequencies, percentages, mean, and median. Initially bivariate logistic regressions were used to model the association of the odds of delayed treatment initiation with clinical severity. Similarly, bivariate logistic regressions were also used to model the association of delayed treatment initiation with the level of infectiousness and then those variables with a *p*-value of at most 0.2 were entered into the multivariate model. A comparison of the proportion of smear-positive tuberculosis and the presence of a pulmonary cavity (level of infectiousness) with the duration of patient delay was modeled using a multivariate logistic regression model. Similarly, the association between clinical severity and the total delay was modeled using multivariate logistic regression. In both logistic regression models the goodness of fit was checked using Hosmer and Lemeshow test. In all analyses, any result with the *p*-value less than 5% was considered as being statistically significant.

### Definition of operational terms

**Lung cavitations**: the presence of at least one cavity on a chest radiograph taken during the episode of TB [31].

**Total delay**: is the time period from the onset of TB symptoms to first start of anti-TB treatment and classified as < 30 days, 31–60 days and > 60 days [34].

**Patient delay**: the time from the onset of symptoms until the first contact with the health care service [34].

**Health system delay**: the time from a patient’s first contact with the health care service until either a TB diagnosis is made or TB treatment is commenced [34].

**Diagnostic delay**: the time from first contact with health care services until a diagnosis is made [34].

## Results

### Demographic characteristics of the study participants

A total of 875 pulmonary tuberculosis patients were included in this study. The median ages of the study participants were 35 years with 25–45 Inter-quartile range (IQR). From the total respondents, 58.1% were males, 54.5% were from urban areas, 27.2% were farmers, 21.6% were housewives, 18.9% were employed, 10.4% were daily laborer, 11.4% were of students and the remaining 10.5% were Unemployed. In terms of socio-economic status, 37.7% were poor (living in debt), 35.6% had income equal to their expense (had medium income) and the remaining 25.8% earned for covering their expenses and savings. With regard to education, 39.9% were illiterate, 37.1% primary school (1–8 grade), 12.5% secondary school (9–12 grade) and 10.5% completed college and above. Regarding marital status, 55.1% were married, 8% were divorced or widowed and 26.7% were single.

### Self-reported clinical symptoms and other clinical profiles

The major self-reported clinical symptoms were cough (99.2%), and fever (48.6%).Of all the study participants 28.7 and 26.6% had a BMI of below 18 and 16, respectively. Similarly, 39.6% had MUAC below 220 mm and 23.5% had MUAC measurement of below 200 mm. The clinical score of 50.9 of the study participants was higher than eight and 51.7% of the study participants had alower level of infectiousness (Table 1). Regarding measures of levels of infectiousness, 295(33.7%) were x-ray^+^ and AFB ^+^, 25(2.9%) x-ray^−^ and AFB ^+^, 103(11.8%) AFB^−^ and x-ray^+^, 452(51.7%) AFB^−^ and x-ray^−^. Concerning the diagnosis of the study participants, 36.6% were AFB positive, 88.2% were GeneXpert positive and 45.5% had suggestive X-ray (like cavitations in the lung).

**Table 1:** self-reported clinical symptoms and other clinical profile of new adult pulmonary tuberculosis patients attending selected health facilities of two zones of Tigray, Northern Ethiopia, 2019 (*n* = 875)

Variable Categories		Frequency	Percent
Symptoms	Cough	868	99.2
	Fever	425	48.6
	Chest pain	389	44.5
	Weight loss	398	45.5
	Haemoptysis	204	23.3
	Others^a^	449	51.3
Presence of other chronic diseases^b^ (excluding HIV)	Yes	23	2.6
	No	852	97.4
HIV status	Positive	99	11.3
	Negative	776	88.7
BMI	Normal (> 18)	391	44.7
	16–18	251	28.7
	< 16	233	26.6
MUAC (in MM)	Normal (> 220)	323	36.9
	200–220	346	39.6
	< 200	206	23.5
Clinical score	Mild (< 8)	430	49.1
	Severe (> 8)	445	50.9
Level of infectiousness	Lower	452	51.7
	Higher	423	48.3

^a^Sweating, loss of appetite, fatigue, chill, malaise ^b^ diabetes, arthritis, epilepsy, chronic liver disease, Visceral leishmaniasis, COPD; BMI (Body Mass Index); MUAC (Mid-Upper Arm Circumference)

### Behavioral and environmental factors of the study participants

Significant proportion of the study participants had history of alcohol intake and lives in crowed situation (large family living in small number of rooms) and smoking, respectively. Prevalence of reporting history of smoking and contact with known TB patients is not very large (Table 2).

**Table 2:** Behavioral and environmental factors of new adult pulmonary tuberculosis patients attending selected health facilities of two zones of Tigray, Northern Ethiopia, 2019 (*n* = 875)

Variable categories		Frequency	Percent
Alcohol use	Yes	464	53
	No	411	47
History of smoking	Yes	89	10.2
	No	786	89.8
Previous TB contact	Yes	128	13.3
	No	747	77.8
Family size	1–3	477	54.5
	> 3	398	45.5
Number of rooms	< 2	358	40.9
	< 2	517	59.1

### Association of TB treatment initiation delay with clinical severity and level of infectiousness

#### The association of TB treatment initiation delay with clinical severity

The median total delay of the study participants was 62 days with an inter-quartile range of 16–221 days. Patients took a median of 30 days to first visit a health provider after the onset of their cough. While the median number of days patients delay in the health system was 18 days; of this health system delay 15 (median) days were due to delay in diagnosis. Of all the study participants, 26.2% came to a health facility and got diagnosis and treatment within 30 days and of those who delayed for more than 60 days, 80% had a clinical score of eight and above.

In multivariate logistic regression analysis (Table 3), those who had not delayed initiating treatment (Adjusted odds ratio (AOR) = 0.04, 95% CI 0.04–0.11) and those who have initiated treatment after a medium delay or from 30 to 60 days (AOR = 0.17, 95% CI 0.06–0.15) were significantly associated with a/the decreased clinical score or mild disease compared to those who initiated treatment after a/the delay for more than 2 months.

**Table 3:** Clinical severity and associated factors among new adult pulmonary tuberculosis patients attending selected health facilities of two zones of Tigray, Northern Ethiopia, 2019 (*n* = 875)

Variables	Categories	Clinical severity		Crude odds ratio	Adjusted odds ratio
		**Mild (< 8)**	**Severe (>8)**		
Sex					
	Female	186	244	0.68(0.81–1.4)	0.9(0.64–1.27)
	Male	181	264	1.00	1.00
Age					
	18–34	211	191	0.58(0.36–0.94)	0.58(0.32–1.03)
	35–44	110	108	0.57(0.34–0.95)	0.61(0.33–1.13)
	45–54	75	96	0.64(0.38–1.1)	0.55(0.29–1.03)
	> 55	34	50	1.00	1.00
Income					
	Indebt	118	210	1.4(1.01–2.05)	1.3(0.86–2.03)
	Income = expense	158	163	1.2(0.85–1.61)	0.96(0.65–1.4)
	Saving	154	72	1.00	1.00
Delay in days					
	No delay (< 30)	233	32	0.07(0.05–0.11)*	0.04(0.04–0.11)*
	Medium delay (31–60)	112	74	0.09(0.06–0.15)*	0.17(0.06–0.15)*
	Long delay (> 60)	85	339	1.00	1.00
HIV status					
	Positive	52	47	0.91(0.59–1.4)	0.91(0.54–1.54)
	Negative	378	398	1.00	1.00
Smoking history					
	Yes	41	48	0.91(0.50–1.42)	1.4(0.81–2.41)
	No	389	398	1.00	1.00
Alcohol consumption					
	Yes	232	232	0.76(0.58–0.99)	0.84(0.61–1.17)
	No	198	213	1.00	1.00

*Indicates statistically significant variables with *P*-value less than 0.05

#### The association of TB treatment initiation delay with the level of infectiousness

In multivariate logistic regression analysis (Table 4), the level of infectiousness was higher in a group that delayed for 30–60 days (AOR = 4.2, 95% CI, 2.82–6.01) and for those who delayed for more than 60 days (AOR = 6.2, 95% CI 4.32–8.83,) as compared to patients who have initiated treatment within 1 month. Similarly, having more than 3 family members has a higher level of infectiousness (AOR = 1.6, 95% CI 1.16–2.14) compared to those who have at most three family members. Whereas, having two and above a number of rooms and being HIV negative were more likely to present with a lower level of infectiousness (AOR = 0.55, 95% CI 240 0.33–0.61) and (AOR = 0.43, 95% CI 0.36–0.91) compared to those who have one room and HIV positive TB patients, respectively.

**Table 4:** Level of infectiousness among new adult pulmonary tuberculosis patients attending selected health facilities of two zones of Tigray, Northern Ethiopia, 2019 (*n* = 875)

Variables	Categories	Level of infectiousness		Odds ratio (OR)	
		Higher	Lower	Crude OR	Adjusted OR
Sex	Male	251	257	1.00	1.00
	Female	172	195	1.1(0.85–1.54)	0.8(0.59–1.31)
Age	18–34	201	201	1.00	1.00
	35–44	100	118	1.1(0.66–1.71)	0.63(.35–1.15)
	45–54	79	92	1.2(0.52–1.7)	1.1(0.61–1.92)
	> 55	43	41	1.2(0.72–2.1)	1.2(0.68–2.16)
Educational status	No formal education	178	171	1.00	1.00
	Primary education	163	162	0.65(0.41–1.03)	0.4(0.22–0.81)
	Secondary education	45	64	0.69(0.43–1.1)	0.7(0.38–1.3)
	College and above	37	55	0.96(0.54–1.68)	0.9(0.52–1.93)
Family size	1–3	196	202	1.00	1.00
	> 3	225	252	1.07(0.83–1.42)	1.6(1.16–2.14)*
Number of rooms	< 2	212	146	1.00	1.00
	> 2	209	308	0.47(0.36–0.62)	0.45(0.33–0.61)*
HIV status	Positive	57	42	1.00	1.00
	Negative	366	410	0.65(0.43–0.99)	0.57(0.36–0.91)*
Delay in days	No delay (< 30)	68	197	1.00	1.00
	Medium delay (31–60)	68	118	3.7(2.56–5.29)*	4.2(2.82–6.01)*
	Long delay (> 60)	287	137	6(4.26–8.46)*	6.2(4.32–8.83)*
Smoking history					
	Yes	23	66	1.78(0.78–3.42)	1.26(0.69–2.29)
	No	390	396	1.00	1.00
Alcohol consumption					
	Yes	222	242	1.76(0.88–2.99)	1.17(0.79–1.72)
	No	210	201	1.00	1.00
Access to health facility	< 10	240	258	0.95(0.49–1.54)	0.92(0.51–1.61)
	> 10	193	182	1.00	1.00

*Indicates statistically significant variables with *P*-value less than 0.05

## Discussion

In the current study, there is a significant association of delayed treatment initiation with clinical severity. This brings the patient to have severe clinical symptoms like hemoptysis which may lead to patient hospitalization, extensive disease, and finally death. This is in line with previous studies in Guinea Bissau [27, 35, 36], Ghana [37], Italy [38] and Spain [39]. In Guinea Bissau [27, 35, 36] the proportion of clinical severity was higher among patients who had long and very long delays. The study in Ghana [37] indicated that a higher risk of hospitalization for TB patients was associated with longer treatment delay. The studies in Italy [38] and Spain [39] shown that delayed TB patients to initiate treatment had resulted in more severe clinical presentation and extensive disease conditions. This might be because the bacteria had got time to multiply, which would lead to further infection and this also allows the MTB to continue growing and overwhelm the cells it has infected which would be resulted in worse clinical symptoms like the presence of a mixture of blood and sputum in the lungs over time [40]. This implies the need for interventions targeted to those delayed treatment initiated patients while they are on follow up.

Compared with patients who initiated treatment within 1 month, the odds of patients presenting with smear-positive tuberculosis and the pulmonary cavity or level of infectiousness was higher among patients who delayed treatment initiation beyond a month. Thus, the presence of a higher proportion of smear positivity and lung cavitations among delayed TB patients indicates that delayed TB patients had a higher level of infectiousness which may lead to increased risk of MTB transmission. This finding is consistent with other studies from the USA [41] and China [28] in which delay was associated with increased smear positivity and lung cavitations. Other previous studies [31, 32] had also shown that TB patients with smear positivity and lung cavitations had a higher risk of MTB transmission compared to smear-negative and those who had no lung cavitations due to a higher level of infectiousness. This could be due to a long duration of delay that would affect the number of bacilli in the lung and sputum which leads to a higher reproductive rate of the bacteria. Studies had indicated status and grade of sputum smear, lung cavitations and time of treatment initiation determine the infectiousness of the index cases [42, 43]. This is by the reason that the number of bacilli found in the sputum of smear-positive patients makes the patients more infectious [14, 44]. This implies that policymakers should design the way families of TB patients could be screened regularly.

In this study HIV positive patients had a higher rate of infectiousness which may lead to a higher risk of MTB transmission compared to HIV negative TB patients. This study was similar to studies from Kenya [45] and the US [41] but contrary to Tanzania [46]. A study in Kenya and the US indicated that the risk of TB infection was increased with increasing HIV prevalence over time and in Tanzania the TB annual risk of infection remained unchanged despite an increase in HIV prevalence [45, 47]. Furthermore; contrary to our findings studies had also indicated a lower rate of smear positivity and lung cavitations among HIV positive patients due to the absence of delayed-type hypersensitivity response [48, 49]. The observed difference or contrary might be due to all of HIV infected patients participated in this study who had started antiretroviral therapy which could improve immune status of the patients and this leads to increased lung cavitations**.**

The findings of the current study should be interpreted with the following limitations. The duration of treatment delay may be influenced by recall bias which may affect the magnitude of patient delay. An effort was made to minimize the bias using different methods. Firstly, we have used local calendars of holidays, special events (marriage, death, birth date and epiphany) which enable the patients to remember the exact date. Second, the study participants were recruited and interviewed immediately after their diagnosis. Furthermore, we do not believe that this bias would affect the association between sputum smear positivity, having a pulmonary cavity and clinical severity with increasing duration of treatment delay as it would be the same for all patients. Rather than using actual disease transmission measures, we used two proxy measures which could potentially bring a worry. However, these two measures have been well associated with TB transmission and have been used by previous studies to measure the risk of MTB transmission [28, 31, 32]. Finally, we did not collect data regarding habit of diet which might have a confounding effect on household crowding and level of infectiousness but the indirect measures of diet like BMI and MUAC were measured and had no effect.

## Conclusion and recommendations

Delayed initiation of TB treatment increases clinical severity and level of infectiousness (i.e, increased smear positivity and lung cavitations). Furthermore; the level of infectiousness was higher among HIV positive TB patients. These findings imply that the regional government should work to reduce the long-delayed treatment initiation through strengthening its diagnostic efficiency and implementation of rapid diagnostic tools at the primary health care units. Delayed and non-delayed patients should be identified during treatment initiation for better clinical management during the treatment follow up period. Future study on the same topic should use real transmission measures so that it addresses one of the limitations of the current study.

## Data Availability

All data is contained in the manuscript and other raw data will be provided to readers upon the request to the corresponding author.
